# Not All That Glows Is Ischaemia: Extensive Hepatic and Mesenteric Venous Gas From Sigmoid Diverticulitis

**DOI:** 10.7759/cureus.91483

**Published:** 2025-09-02

**Authors:** Sandeepa D Dadigamuwage, Vimarshini Samarakoon, Marlon Brown, Sreeranj Madathiparambil, Rajesh Kochupapy

**Affiliations:** 1 Colorectal Surgery, University Hospitals Plymouth NHS Trust, Plymouth, GBR; 2 Cardiothoracic Surgery, University Hospitals Plymouth NHS Trust, Plymouth, GBR

**Keywords:** computed tomography, conservative treatment, gas-forming organisms, hepatic portal venous gas, mesenteric venous gas, non-operative management, sigmoid diverticulitis

## Abstract

Hepatic portal venous gas (HPVG) is a rare radiological finding most commonly associated with bowel ischaemia and high mortality. However, it can also occur in benign conditions such as diverticulitis, where the clinical course may be less severe. Management decisions must balance the alarming imaging appearance with the patient’s clinical stability. We present the case of a 47-year-old male patient with underlying diverticular disease who developed extensive HPVG and mesenteric venous gas secondary to sigmoid diverticulitis. Despite the dramatic radiological findings, he remained haemodynamically stable with no signs of peritonism. Blood cultures grew *Pseudomonas aeruginosa *and *Escherichia coli*, suggesting a gas-forming infective source. He was managed conservatively with intravenous antibiotics and close monitoring. Repeat imaging showed a significant reduction in portal venous gas, and the patient made a full recovery without requiring surgery. He was discharged on day 9 with outpatient follow-up for elective sigmoid colectomy. This case highlights that HPVG, while often considered a surgical emergency, may be managed non-operatively in selected patients with diverticulitis who are clinically stable. Multidisciplinary evaluation and careful correlation between imaging and clinical findings are essential in guiding appropriate management.

## Introduction

Hepatic portal venous gas (HPVG) is an uncommon but clinically significant radiological finding, often associated with severe intra-abdominal pathology such as bowel ischaemia, with mortality rates historically reported to be as high as 75% [1]. Although ischaemia remains the most frequent cause, HPVG has been increasingly identified in association with other intra-abdominal conditions, including inflammatory bowel disease, intra-abdominal sepsis, bowel obstruction, and iatrogenic interventions [2,3].

Diverticulitis is a common inflammatory disease of the colon, particularly affecting the sigmoid segment in Western populations. While HPVG secondary to diverticulitis is rare, its occurrence raises concern for complicated or perforated disease, especially when associated with mesenteric venous gas or septic thrombophlebitis [4,5]. Gas may enter the portal circulation via transmural migration through inflamed mucosa or from gas-forming organisms in the presence of bacteraemia or abscess formation [3,6].

With the growing use of contrast-enhanced computed tomography (CT), HPVG is now more frequently detected in cases that are clinically stable and not necessarily indicative of bowel infarction [7]. Emerging evidence suggests that non-operative management may be appropriate in selected patients, provided close clinical and radiological monitoring is ensured [5,8]. We report a case of extensive HPVG and mesenteric venous gas in a patient with sigmoid diverticulitis who remained haemodynamically stable and was successfully treated with conservative management.

## Case presentation

A 47-year-old male patient with a background of hypertension, hypercholesterolaemia, exertional chest pain under investigation for coronary artery disease, gastro-oesophageal reflux disease, and a strong family history of cardiovascular disease initially presented to his general practitioner with a one-week history of left elbow pain, erythema, and swelling. He was treated empirically for olecranon bursitis with oral flucloxacillin.

On day 1, the patient developed fever with chills, rigours, and generalised fatigue and was brought to the emergency department by ambulance. Clinical examination revealed a low-grade fever (38.4°C) with mild left elbow swelling and tenderness. Initial haematological investigations are summarised in Table 1, and venous blood gas (VBG) analysis in Table 2. He was discharged with a working diagnosis of resolving olecranon bursitis.

**Table 1:** Haematological investigations on admission eGFR: estimated glomerular filtration rate; CRP: C-reactive protein; ALP: alkaline phosphatase; ALT: alanine transaminase; WCC: white cell count; HB: haemoglobin

Parameter	Results	Reference range
eGFR	>90	>90 ml/min/1.73 m^2^
CRP	27	0.1-5 mg/L
Sodium	139	133-146 mmol/L
Potassium	4.3	3.5-5.3 mmol/L
Urea	6.9	2.5-7.8 mmol/L
Creatinine	81	64-104 mmol/L
Albumin	42	35-50 g/L
ALP	58	30-130 IU/L
Bilirubin	19	1-20 µmol/L
ALT	41	1-55 IU/L
Troponin I	14	0-34 ng/L
WCC	13.2	3.6-9.2 x 10^9^/L
HB	160	130-175 g/L
Platelets	173	150-450 x 10^9^/L
Neutrophils	12.2	1.7-6.2 x 10^9^/L

**Table 2:** Blood gas analysis on admission PaO2: partial pressure of arterial oxygen; PaCO2: partial pressure of carbon dioxide in arterial blood; HCO3: bicarbonate

Parameter	Results	Reference range
PH	7.42	7.35-7.45
PaO2	3.96	10-13 kPa
PaCO2	5.54	4.7-6.0 kPa
HCO3	26.9	22-26 mmol/L
Lactate	1.2	0.5-2.0 mmol/L

Blood cultures obtained during the initial admission later grew *Pseudomonas aeruginosa *and *Escherichia coli*. Two days later, the patient re-presented to the emergency department with new suprapubic and left iliac fossa pain. Examination revealed mild left lower quadrant tenderness without signs of peritonism. Repeat laboratory results on admission are shown in Table 3, with a marked inflammatory response, mild thrombocytopenia, and liver function derangement.

**Table 3:** Haematological investigations on re-admission eGFR: estimated glomerular filtration rate; CRP: C-reactive protein; ALP: alkaline phosphatase; ALT: alanine transaminase; WCC: white cell count; HB: haemoglobin

Parameter	Results	Reference range
eGFR	80	>90 ml/min/1.73 m^2^
CRP	225	0.1-5 mg/L
Sodium	138	133-146 mmol/L
Potassium	4.1	3.5-5.3 mmol/L
Urea	9.1	2.5-7.8 mmol/L
Creatinine	97	64-104 mmol/L
Albumin	37	35-50 g/L
ALP	96	30-130 IU/L
Bilirubin	57	1-20 µmol/L
ALT	106	1-55 IU/L
Troponin I	8	0-34 ng/L
WCC	6.3	3.6-9.2 x 10^9^/L
HB	156	130-175 g/L
Platelets	114	150-450 x 10^9^/L
Neutrophils	5.6	1.7-6.2 x 10^9^/L

**Figure 1:**
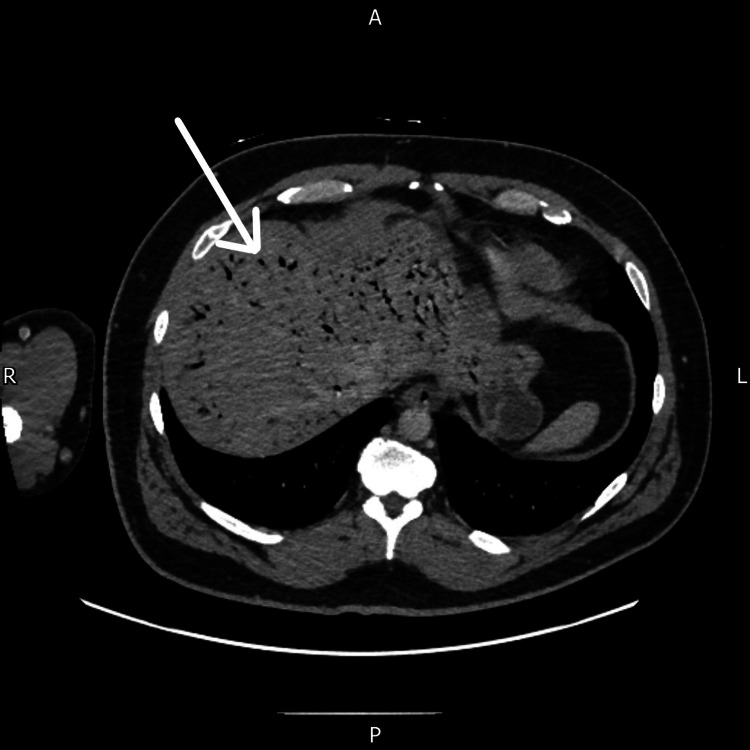
CT scan of the abdomen and pelvis showing extensive gas accumulation within the hepatic-portal vein branches, as observed in the axial view

**Figure 2:**
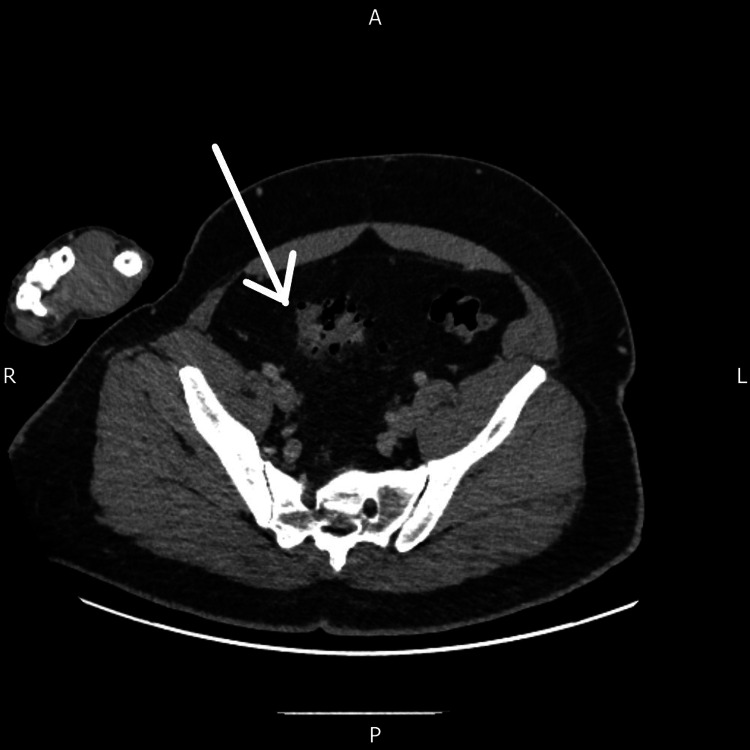
CT scan of the abdomen and pelvis demonstrating inflammatory changes in the mid sigmoid colon surrounding diverticula, suggestive of diverticulitis, seen in the axial view

A contrast-enhanced CT scan of the abdomen and pelvis revealed extensive portal venous gas in the superior segments of the liver (Figure 1), along with gas within the mesenteric venous system. Acute sigmoid diverticulitis with localised inflammatory changes was identified (Figure 2), with no evidence of bowel ischaemia, perforation, or intra-abdominal collection. Based on these radiological findings, the case was classified as modified Hinchey stage 1a (confined pericolic inflammation).

He was admitted under general surgery, commenced on intravenous piperacillin-tazobactam 4.5 g four times daily and remained haemodynamically stable. The patient’s National Emergency Laparotomy Audit (NELA) risk score was calculated at 0.88%, indicating a low estimated 30-day mortality risk should emergency surgery have been required. Given the absence of peritonism and the localised nature of the disease, a decision was made to pursue non-operative management with supportive care. The patient was maintained nil by mouth (NPO) initially with intravenous hydration and antibiotics, followed by gradual reintroduction of oral intake on day 3 as symptoms improved.

**Figure 3:**
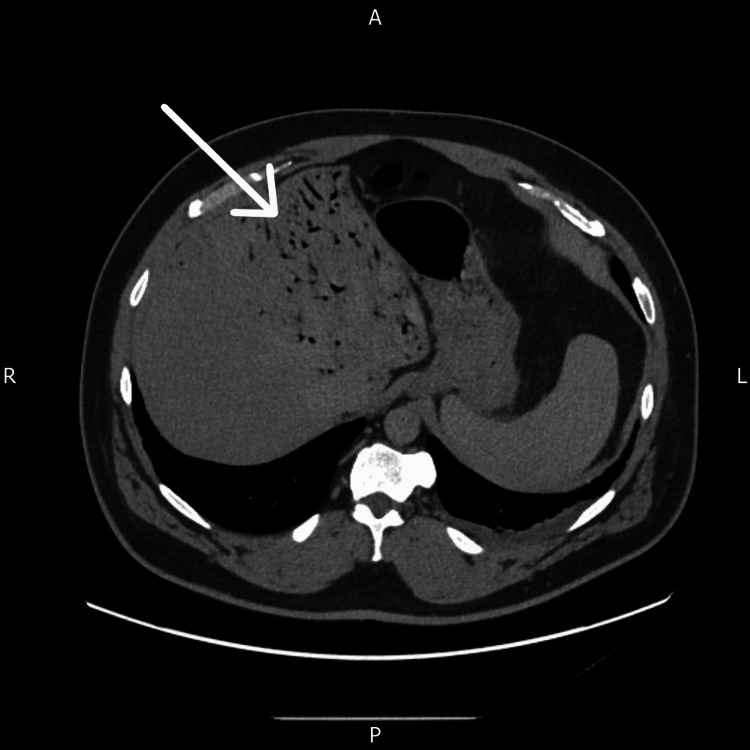
Repeat axial CT scan of the abdomen showing a reduced volume of portal venous gas within the left lobe of the liver

After 48 hours of treatment, inflammatory markers improved substantially (Table 4), and a repeat contrast-enhanced CT scan demonstrated a significant reduction in HPVG, now predominantly confined to the left hepatic lobe (Figure 3). Mesenteric gas persisted but showed no progression. The sigmoid diverticulitis remained radiologically stable, with no evidence of perforation, abscess formation, or new intra-abdominal collections. The patient remained haemodynamically stable throughout admission, with no features of sepsis or clinical deterioration. His abdominal symptoms gradually resolved with supportive care.

**Table 4:** Improved haematological investigations following conservative management eGFR: estimated glomerular filtration rate; CRP: C-reactive protein; ALP: alkaline phosphatase; ALT: alanine transaminase; WCC: white cell count; HB: haemoglobin

Parameter	Results	Reference range
eGFR	>90	>90 ml/min/1.73 m^2^
CRP	130	0.1-5 mg/L
Sodium	139	133-146 mmol/L
Potassium	3.5	3.5-5.3 mmol/L
Urea	7.5	2.5-7.8 mmol/L
Creatinine	81	64-104 mmol/L
Albumin	30	35-50 g/L
ALP	115	30-130 IU/L
Bilirubin	53	1-20 µmol/L
ALT	81	1-55 IU/L
WCC	6.7	3.6-9.2 x 10^9^/L
HB	141	130-175 g/L
Platelets	110	150-450 x 10^9^/L
Neutrophils	5.0	1.7-6.2 x 10^9^/L

A multidisciplinary decision supported continued non-operative care given clinical and radiological improvement, and patient-specific surgical risks. He was gradually reintroduced to oral intake and made an uneventful recovery. Repeat blood cultures performed at discharge showed no growth, confirming clearance of bacteraemia.

He was discharged home on day 9, with outpatient follow-up arranged to plan for colonoscopy and elective sigmoid colectomy. Haematological investigations at the time of discharge are summarised in Table 5.

**Table 5:** Haematological investigations on discharge eGFR: estimated glomerular filtration rate; CRP: C-reactive protein; ALP: alkaline phosphatase; ALT: alanine transaminase; WCC: white cell count; HB: haemoglobin

Parameter	Results	Reference range
eGFR	>90	>90 ml/min/1.73 m^2^
CRP	33	0.1-5mg/L
Sodium	140	133-146 mmol/L
Potassium	4.1	3.5-5.3 mmol/L
Urea	4.0	2.5-7.8 mmol/L
Creatinine	75	64-104 mmol/L
Albumin	33	35-50 g/L
ALP	297	30-130 IU/L
Bilirubin	18	1-20 µmol/L
ALT	57	1-55 IU/L
WCC	10.7	3.6-9.2 x 10^9^/L
HB	138	130-175 g/L
Platelets	227	150-450 x 10^9^/L
Neutrophils	7.4	1.7-6.2 x 10^9^/L

## Discussion

HPVG is historically associated with catastrophic abdominal events such as mesenteric ischaemia or bowel necrosis, where emergent surgery is typically warranted. However, the increasing use of high-resolution CT has broadened our understanding of HPVG, revealing that it can occur in a wider range of pathologies, including inflammatory, infectious, and iatrogenic causes, without always portending a poor prognosis [1-3].

Diverticulitis-associated HPVG is a rare but documented entity. Sellner et al. reviewed 21 cases of HPVG related to sigmoid diverticulitis and found that outcomes differed significantly depending on the underlying mechanism: patients with mesocolic abscesses had more favourable outcomes than those with septic thrombophlebitis [5]. In our case, the patient had extensive HPVG and mesenteric venous gas on imaging but showed no radiological evidence of ischaemia or abscess formation and was haemodynamically stable with no signs of peritonism. These features were pivotal in deciding against immediate surgical intervention.

Several studies support a selective, non-operative approach in patients with HPVG who lack overt signs of sepsis or bowel compromise [4,6-8]. Wayne et al. proposed a decision algorithm where surgery is reserved for patients with clinical deterioration or radiological signs of infarction [9]. Kinoshita et al. similarly emphasised that HPVG in stable patients should be interpreted in conjunction with clinical and laboratory data, rather than in isolation [2].

The pathophysiology of HPVG in diverticulitis is likely multifactorial. In our patient, positive blood cultures for gas-forming organisms (*Pseudomonas aeruginosa *and* Escherichia coli*) suggest bacterial translocation into the portal venous system, potentially via phlebitis in the inferior mesenteric vein. CT findings of surrounding stranding in the venous branches support this mechanism. However, there was no pneumatosis intestinalis, no portal vein thrombosis, and no fluid collections, further supporting conservative management. Additionally, the patient’s preceding episode of olecranon bursitis may have served as a remote source of bacteraemia, adding to the complexity of the case.

Importantly, the radiological extent of HPVG does not always correlate with disease severity. Our patient had extensive portal and mesenteric venous gas, yet only mild localised diverticulitis on CT and a rapid clinical response to antibiotics. This mismatch has been noted in other reports, where the virulence of pathogens and host immune status (e.g., immunocompromised or diabetic patients) may explain disproportionate gas formation [5,10].

Operative intervention in this patient carried a moderate risk, with an uninvestigated history of exertional angina, elevated BMI, and NELA risk of 0.88%. A multidisciplinary discussion with surgery, radiology, and anaesthetics concluded that continued conservative treatment was safest, with surgery reserved for clinical deterioration. This mirrors current literature advocating individualised treatment over algorithmic surgical intervention [9,11-13].

Follow-up imaging demonstrated a marked reduction in hepatic portal gas and no progression of mesenteric gas, confirming the appropriateness of non-operative management. Several case reports have shown similar outcomes, with complete radiological resolution following conservative therapy in the absence of ischaemia [6,8,14]. It is important to note that many studies on HPVG involve a heterogeneous group of aetiologies, and only a limited number specifically focus on diverticulitis. This limits the direct applicability of existing evidence to our case and underscores the need for cautious interpretation.

This case reinforces the growing view that HPVG is a radiological sign, not a diagnosis, and that surgical decisions should be based on clinical context, not radiological findings alone. A measured, evidence-based approach can prevent unnecessary surgery in carefully selected patients.

## Conclusions

Hepatic-portal and mesenteric venous gas is an alarming radiological sign traditionally associated with serious pathology such as bowel ischaemia or necrosis. However, with the increasing use of cross-sectional imaging, it is now recognised in a broader range of conditions, including diverticulitis. This case demonstrates that even extensive HPVG can occur in the absence of peritonism, ischaemia, or systemic instability, and does not always necessitate emergency surgery.

Our patient remained clinically stable despite the dramatic imaging findings and responded well to conservative management. This highlights the importance of a patient-centred, multidisciplinary approach that considers the full clinical picture rather than relying solely on radiological severity. Carefully selected patients with diverticulitis-associated HPVG can be safely managed without operative intervention, avoiding unnecessary surgical morbidity.
